# Effects of Plant-Based Diets on Weight Status in Type 2 Diabetes: A Systematic Review and Meta-Analysis of Randomised Controlled Trials

**DOI:** 10.3390/nu13114099

**Published:** 2021-11-16

**Authors:** Grace Austin, Jessica J. A. Ferguson, Manohar L. Garg

**Affiliations:** 1 Nutraceuticals Research Program, School of Biomedical Sciences & Pharmacy, University of Newcastle, 305C, Medical Science Building, Callaghan, NSW 2308, Australia; grace.austin@uon.edu.au (G.A.); jessica.ferguson@newcastle.edu.au (J.J.A.F.); 2 Hunter Medical Research Institute, University of Newcastle, New Lambton, NSW 2035, Australia

**Keywords:** plant-based diet, vegan, vegetarian, pescatarian, pesco-vegetarian, lacto-ovo-vegetarian, weight, BMI, waist circumference

## Abstract

Excessive adiposity is a major risk factor for type 2 diabetes (T2D), and dietary patterns are important determinants of weight status. Plant-based dietary patterns (PBDs) are known for their therapeutic effects on T2D. The aim is to systematically review RCTs to investigate the effects of various PBDs compared to regular meat-eating diets (RMDs), in individuals who normally consume a RMD on body weight, BMI, and waist circumference in T2D. RCTs investigating PBDs and body weight, BMI, WC for ≥6 weeks in adults with T2D since 1980 were eligible for inclusion. Seven trials (*n* = 269) were included in the meta-analysis using random-effects models and expressed as MD (95%Cls). Compared to RMDs, PBDs significantly lowered body weight (−2.35 kg, 95% CI: −3.51, −1.19, *p* < 0.001), BMI (−0.90 kg/m^2^, 95% CI: −1.42, −0.38, *p* = 0.001) and WC (−2.41 cm, 95% CI: −3.72, −1.09, *p* < 0.001). PBDs alone significantly reduced body weight by 5.1% (−4.95 kg, 95% CI: −7.34, −2.55, *p* < 0.001), BMI by 5.4% (−1.87 kg/m^2^, 95% CI: −2.78, −0.95, *p* < 0.001) and WC by 4.3%(−4.23, 95% CI: −6.38, −2.07, *p* < 0.001). Interventions not limiting energy intake led to a significant reduction in body weight (−2.54 kg, 95% CI: −4.16, −0.92, *p* < 0.005) and BMI (−0.91 kg/m^2^, 95% CI: −1.56, −0.25, *p* < 0.005). Trials ≥16 weeks had a pronounced reduction in body weight (−2.93 kg, 95% CI: −5.00, −0.87, *p* = 0.005) and BMI (−1.13 kg/m^2^, 95% CI: −1.89, −0.38, *p* < 0.005). These findings provide evidence for the implementation of PBDs for better management of central adiposity in individuals with T2D.

## 1. Introduction

Type 2 diabetes (T2D) is recognised as the fastest growing chronic condition across the globe [1]. According to the 2015 International Diabetes Federation Diabetes Atlas, one in every 11 adults had diagnosed diabetes, 90% of whom had T2D [2]. Independent of an individual’s genetic disposition towards T2D, excessive adiposity is a dominant factor for increased risk well as other modifiable factors including insufficient physical activity, hypertension, energy-dense diets and overweight/obesity [3]. Furthermore, the global trend of escalating body weight is in parallel with increasing prevalence in developed countries [2,4].

The World Health Organisation states T2D can be treated and its consequences avoided or delayed with diet, physical activity and medication [2,5]. ‘Diabetes Australia Best Practice Guidelines’ for T2D management includes a diet aligned with the Australian Dietary Guidelines and a 5–10% weight loss for overweight or obese individuals [6]. It has been reported that lifestyle interventions including a low-calorie diet and at least 150 min of exercise per week is more effective at lowering incidence of T2D by 58% than oral hypoglycaemic medications such as metformin, only 31% [7].

Diets rich in whole grains, fruits, vegetables, legumes, and nuts; moderate in alcohol consumption; and low in refined grains, red/processed meats, and sugar-sweetened beverages have been shown to reduce T2D risk and improve management of glycaemic indices and blood lipids in individuals with T2D. Examples include, The Mediterranean Diet, Dietary Approaches to Stop Hypertension, low-carbohydrate diet, Alternative Healthy Eating Index approach, Prudent Pattern and vegan and vegetarian dietary patterns [8]. However, maintaining a healthy body weight and dietary pattern continues to be a great challenge in the modern obesogenic environment [5].

In recent years, there has been a steady increase in the awareness and popularity of plant-based diets (PBD) [9]. This is due to the growing research into the health effects such as weight reduction, concerns for animal welfare and ethics, environmental sustainability, perceived healthiness, and the overall positive perception by the public [9,10]. PBDs include dietary patterns that are characterised by a high emphasis on consumption of plant foods and low intakes of animal flesh and/or animal-derived products [11]. PBDs encompass a diverse group of dietary patterns with the most common being: vegan (nil animal products), lacto-ovo vegetarian (including dairy products and eggs), pesco-vegetarian (including fish/seafood with/without dairy and eggs) and semi-vegetarian (minimal and/or infrequent meat consumption).

PBDs have been studied for their preventative and therapeutic effects on T2D and believed to be more beneficial than medication for management [12,13]. Several systematic reviews report the substantial health benefits obtained from following a PBD including a reduction in body weight, blood pressure, blood cholesterol, obesity-related inflammatory markers and reducing the risk of T2D and CVD mortality [11,14,15,16,17,18]. Furthermore, a number of systematic reviews and meta-analyses of cohort studies demonstrate an association between red meat consumption and elevated T2D risk [19,20] and incidence of both CVD events and mortality rates [21]. Two recent meta-analyses of RCTs in predominately overweight/obese individuals reported that vegetarian diets with no energy restrictions led to significant weight loss (−2.02 kg [22] and −3.4 kg [14]). While PBDs have been proven to be successful in reducing body weight in healthy individuals, a systematic review and meta-analysis investigating their role in reducing body weight and other outcomes such as BMI and waist circumference (WC) specifically in individuals with T2D has not been conducted. Since central obesity and/or weight gain play an important role in the pathophysiology of T2D, there remains a need to design nutritional strategies to prevent, ameliorate, and effectively manage these problems [23]. The aim of this study is to systematically review RCTs to report the effects of various PBDs compared to regular meat-eating diets (RMDs) on body weight, BMI, and WC in individuals with T2D. Findings from this study may provide evidence to support the implementation of PBDs for assisting in better management of body weight in individuals with T2D.

## 2. Materials and Methods

### 2.1. Search Strategy

Cochrane Handbook for Systematic Reviews of Interventions (Version 6.2, 2021) was used for the planning and conduct of this systematic review and meta-analysis [24]. The PRISMA statements, guidelines, and checklist was used for reporting (Table S1 in Supporting Information online). The protocol was registered in the PROSPERO international prospective register of systematic reviews (CRD42021222987). A research librarian assisted with the initial search strategy in November 2020. Four electronic databases were first searched (Cochrane Library, CINAHL, Medline and EMBASE) in Oct 2020, and the search was updated in April 2021. The following Medical Subject Headings (MeSH) terms, words and/or their combinations were searched: body weight*, body mass index, BMI, adipos*, overweight, obes*,obesity, body weight changes, weight gain, weight loss, weight change, diabetes mellitus, diabetes, type 2 diabetes, non-insulin depend*, T2DM, NIDDM, diet vegetarian, plant based, PBD, vegetar*, vegan*, pescatarian, plant-based, clinical studies, clinical trials, controlled clinical trials, randomized controlled trials, observational studies, random*, trial*, intervention*, group, placebo*, prospective*, cohort*, observational*. Keywords were searched in the title and abstract and combined using the Boolean operator ‘AND’. Limits included full text publications from 1980–2021 in humans and adults and English language.

### 2.2. Study Eligibility Criteria

Pre-specified Population, Intervention, Comparator, Outcomes, Study Design (PICOS) criteria were used to select studies for inclusion (Table 1). Non-randomised controlled trials, cross-sectional analyses, retrospective cohort studies, review articles, case control studies, conference abstracts and articles that did not report anthropometric measures (body weight, BMI, WC) as outcomes were ineligible for inclusion. Studies reporting incomplete data for anthropometric measures were excluded if required information could not be retrieved when contacting authors.

### 2.3. Selection Process and Quality Assessment

Two of the authors (GA and JJAF) used Covidence systematic review management software (Melbourne, Victoria, Australia) to screen title and abstracts in duplicate for the first selection process based on the eligibility criteria (Table 1). Any additional records identified through reference lists were included the screening process. Studies with T2DM population that included body weight, BMI or WC outcomes were selected if they involved any type of PBD intervention (e.g., semi-vegetarian, pesco-vegetarian, lacto-ovo vegetarian, vegan) and control group without dietary intervention but inclusion of meat (e.g., RMD). Full text publications of articles that appeared to meet the eligibility screening process were retrieved, and the same two authors undertook a second selection assessment. Any discrepancies in the assessment and/or decision-making of selection were resolved in discussion with a third independent research investigator (MLG).

Methodological quality of selected full texts was assessed using the Quality Criteria Checklist for Primary Research in the American Dietetic Association’s Evidence Analysis Manual [25] by two independent research investigators (GA and JJAF) in duplicate (Table S2 in Supporting Information online). This Quality Criteria Checklist allowed for comprehensive appraisal of the validity and significance of selected publications. The checklist contains 10 structured validity questions and additional sub-questions specific to different types of research designs. The risk of bias and scientific quality was assessed through the following validity criteria: research question; selection of participants; comparability of study groups; withdrawal handling; blinding methods employed (if any); description of intervention, procedures, and intervening factors; definition of outcomes, including validity and reliability of measurements; statistical methodology; conclusions; and biases and limitations considering any funding or sponsorships. An overall systematic and objective rating (i.e., positive, negative, or neutral) was assigned to each publication. A positive rating was given if the study met all priority criteria and most of the validity criteria. Priority criteria specifically address the methodology in relation to participant selection and recruitment; comparability of study groups; provision of adequate detail regarding the intervention and data collection process; use of valid and appropriate measurement tools and/or methods for study outcomes and whether potential confounders were considered. A neutral rating indicates studies that have met most of the validity criteria but have not met ≥1 of the priority criteria, implying that the study is not entirely strong. A negative rating indicates that studies have not met ≥6 of the validity criteria. A third independent researcher (MLG) was involved in discussions to resolve any discrepancies in decision making between the two independent research investigators.

### 2.4. Data Extraction

Data extracted from the studies included study identification (author/s, year published, geographic location, article title, journal); study design (cross-over or parallel, blinding level); study quality; duration; target population; sample size of both intervention and control group; participant characteristics (age, sex, health status); intervention characteristics (PBD category); macronutrient composition; baseline and post-intervention energy intake (kcal/day); differences between and within group energy intake (kcal/day); dietary assessment method and adherence techniques (e.g., 24 h food recalls, 3-day food records); pre-study dietary pattern and screening criterion of dietary pattern groups; funding source; and baseline and post-intervention and/or follow-up data on body weight, BMI, and WC. Means or medians and variance measures such as standard differences (SD), SEM, or 95% CIs were collected where possible. Authors of publications with missing data were contacted as required.

### 2.5. Quantitative Data Synthesis

The meta-analysis was conducted using Comprehensive Meta-Analysis software (version 3; Biostat, Englewood, NJ, USA). If ≥3 or more articles reported body weight, BMI and/or WC they were pooled for meta-analysis. Data included was sample size, mean change, SD of the intervention and control groups post-intervention. For cross-over studies, data post-intervention were included for the two intervention groups. If SEM was reported for variation, it was converted to SD using the following formula: SD = SEM × $\sqrt{n}$. Given the diversity in study design and populations, pooled effect sizes and 95% CIs were calculated using a random effects model, and a fixed effects model was conducted for sensitivity analysis previously described by DerSimonian and Laird [26]. Percentage change for each pooled outcome was calculated by dividing the MD by the median baseline value from the included studies (based on the baseline of both the test and control diets in parallel trials and the baseline of the first arm in crossover trials) to improve clinical translation of the pooled estimates. Heterogeneity between studies was assessed using *I*^2^ statistic (estimated proportion of total variation across studies due to heterogeneity rather than chance variation) and interpreted according to Cochrane recommendations: 0–40% may not be important, 30–60% may represent moderate heterogeneity, 50–90% may represent substantial heterogeneity, and 75–100% may represent considerable heterogeneity [27]. Further subgroup analysis was used to explore probably causes (trial length, PBD type, energy intake). Funnel plots and Egger test were used to assess publication bias, with *p* < 0.05 considered evidence of publication bias. To establish whether any single study excessively influenced the overall results, a one-study-removed sensitivity analysis was conducted using the fixed effect model [28]. Subgroup moderator analysis was conducted based on type of PBD interventions, energy intake, and trial length. As per the Cochrane recommendations, interpretation of subgroup analysis should be considered exploratory as less than 10 trials were available for each subgroup. Effect sizes are presented as MD 95% CIs; findings were considered statistically significant if the 95% CI did not cross the zero-point estimate line and *p* < 0.05.

## 3. Results

### 3.1. Overview of Publications

**Figure 1:**
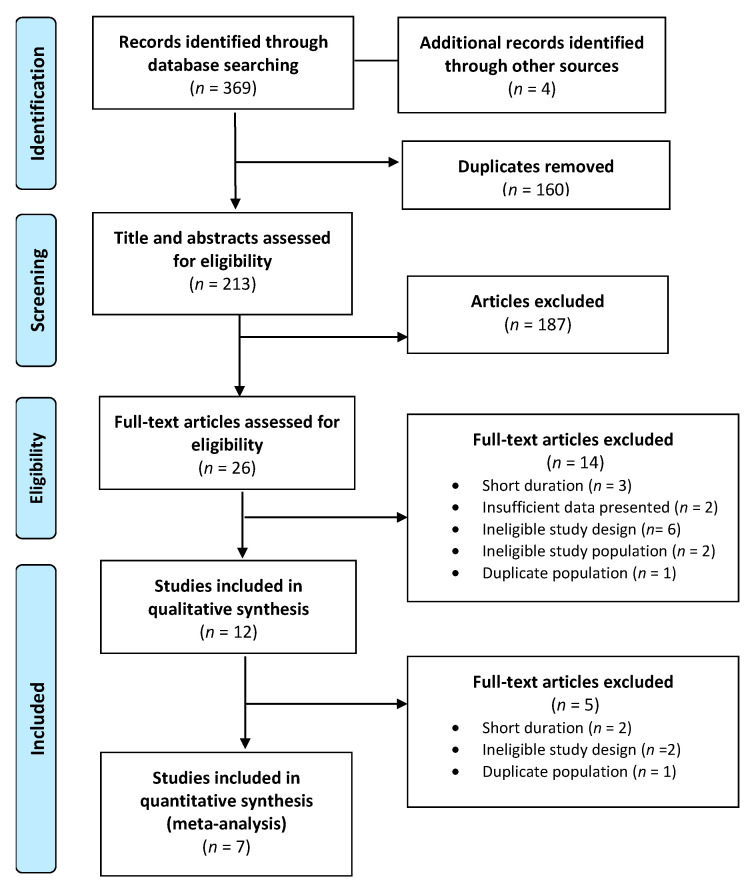
PRISMA flow diagram of study selection.

A total of 369 publications were identified in the database search and 160 duplicates were discarded. Following title and abstract screening, 26 articles met the inclusion criteria, and their full texts were reviewed for eligibility. After further exclusions were applied based on the exclusion criteria, a total of 7 publications were assessed for methodological quality and underwent data extraction. A final total of 7 publications (Figure 1) were included in the systematic review (Table 2) and meta-analysis (Table 3) [29,30,31,32,33,34,35].

### 3.2. Characteristics of Publications

A total of 353 participants with T2D were included in the reviewed publications (Table 2). Trials were conducted in outpatient settings with over two thirds conducted in the United States (*n* = 5, 71%), one from Czech Republic and one from South Korea. All studies were parallel RCTs except for one which was a cross-over and included studies had a median duration of 12 weeks (ranging from 6 to 22 weeks). Prospective cohort studies were eligible for inclusion, however, none meet the inclusion criteria. Participants were equally distributed between males (47.4%) and females (52.6%), typically middle-aged with a mean age of 57.1 years (ranging from 51.0 to 61.0 years) and were obese with a mean BMI of 32.6 kg/m^2^ (ranging from 23.1 to 36.0 kg/m^2^) and a mean WC of 103.2 cm (ranging from 85 to 113.7 cm). The majority (5/7) of studies did not blind the participants.

Six publications included vegan as the PBD intervention, and the remaining publication included lacto-ovo vegetarians. The control groups in all studies were categorised as RMDs because these groups did not restrict or omit meat intake. Six publications specified the exclusion of individuals currently adhering to a vegan or vegetarian diet and the remaining study did not specify; however, it did include a 1-week run-in period [35]. All publications measured energy intake using 2–3-day food records pre and post interventions except for one which used 12 unannounced 24 h food recalls [32]. Dietary advice (*n* = 5) involving nutrition counselling and/or cooking classes were the most common form of intervention delivery followed by meal supplements (*n* = 2) where meals and/or food items were provided with no dietary advice (Table 2). The mean (range) of reported macronutrient intake of intervention groups (*n* = 6) were carbohydrate 65% E (50–75% E), protein 16% E (14–20% E), and fat 19% E (10–30% E) and for the control groups: carbohydrate 56% E (50–70% E), protein 19% E (15–21% E), and fat 27% E (10–35% E). Five studies from the intervention group had no limits on energy intake, and the remaining two studies had adequate and decreased energy intake (Table 2). Within the control groups, there were a mixture of energy intake outcomes, decreased (*n* = 3), adequate (*n* = 2), and not limited (*n* = 2). All seven publications reported weight (kg), five reported BMI (kg/m^2^), and two reported WC (cm).

### 3.3. Study Quality

The majority of the publications (*n* = 6) were categorised as positive, and one was classified as neutral (Table 2 and Table S2 in the Supporting Information online). The studies were funded (*n* = 5) by a government agency, university, or not-for profit organisation with the remaining two not reporting any funding.

### 3.4. Effect of PBDs on Body Weight, BMI, and WC in Individuals with T2D

**Figure 2:**
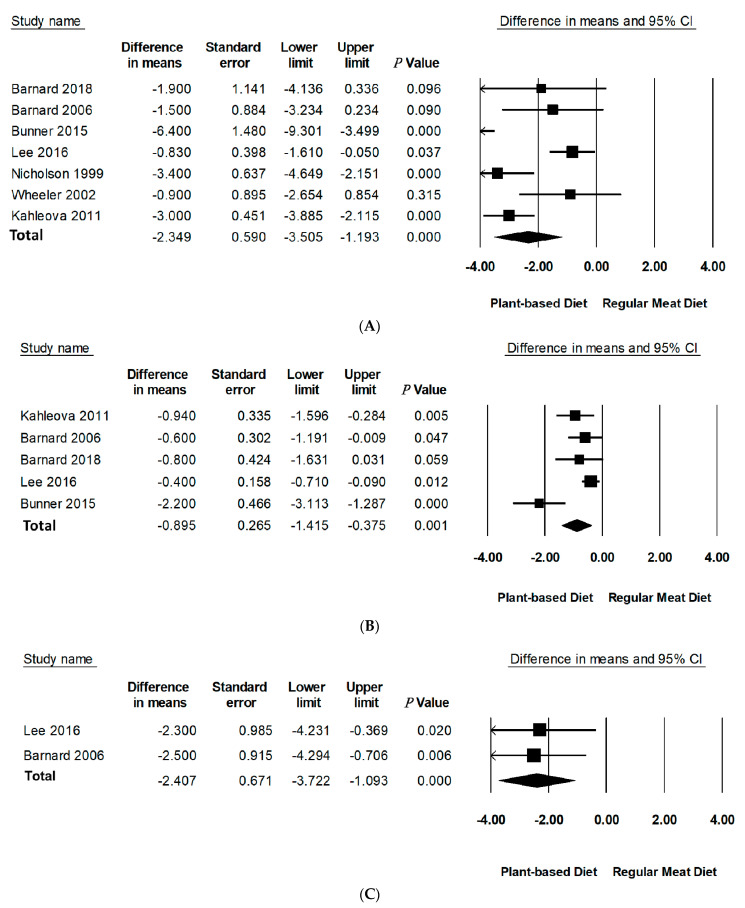
Forest plots displaying difference in means (MD) and 95%Cls for the effect of PBDs compared to RMDs on (**A**) body weight (kg) [30,31,32,33,34,35,36], (**B**) BMI (kg/m^2^) [29,30,32,33,34], and (**C**) waist circumference (cm) [32,34].

Compared to RMDs, PBDs led to a statistically significant reduction in mean differences of body weight (−2.35 kg, 95% CI: −3.51 to −1.19, *p* < 0.001), BMI (−0.90 kg/m^2^, 95% CI: −1.42 to −0.38, *p* = 0.001), and WC (−2.41 cm, 95% CI: −3.72 to −1.09, *p* < 0.001) (Table 3, Figure 2). Specifically, PBDs alone reported a statistically significant reduction in mean differences of body weight (−4.95 kg, 95% CI: −7.34 to −2.55, *p* < 0.001), BMI (−1.87 kg/m^2^, 95% CI: −2.78 to −0.95, *p* < 0.001), and WC −4.23 (95% CI: −6.38 to −2.07, *p* < 0.001). The percentage change was 5.1% for body weight, 5.4% for BMI, and 4.3% for WC. It should be noted that only two studies reported data for WC.

Leave-one-out sensitivity analysis for body weight and BMI showed that these effect sizes were not sensitive to any single study and remained robust for outcomes (Figure S1 in Supporting Information online). A sensitivity analysis could not be performed for WC as there were only two studies with available data. A sensitivity analysis by removal of negative quality studies could not be performed as there were no negative quality studies included in this meta-analysis with only one neutral quality study. There was considerable inter-study heterogeneity for body weight (*I*^2^ = 78.43, *p* < 0.001) and BMI (*I*^2^ = 85.32, *p* < 0.001). Further investigation of methodological diversity reported longer studies to have more heterogeneity than shorter ones (studies over 16 weeks had greater heterogeneity than studies less than 16 weeks, *I*^2^ = 90.93, *p* < 0.001 vs. *I*^2^ = 78.42, *p* < 0.001 accordingly). Analysis of PBD type and energy intake to assess contribution to heterogeneity could not be performed due to the limited number of studies in subgroups.

### 3.5. Publication Bias

There was no evidence to suggest publication bias for weight; however, Eggers linear regression revealed statistically significant publication bias for BMI (intercept, −8.05, SE, 1.12; 95% CI, −11.64 to −4.48; t = 7.16, df, 3; 2-tailed *p* < 0.005) (Figure S1 in the Supporting Information online). A publication bias analysis for WC could not be performed as procedures require at least three studies, and there were only two with available data.

### 3.6. Subgroup Analyses

**Figure 3:**
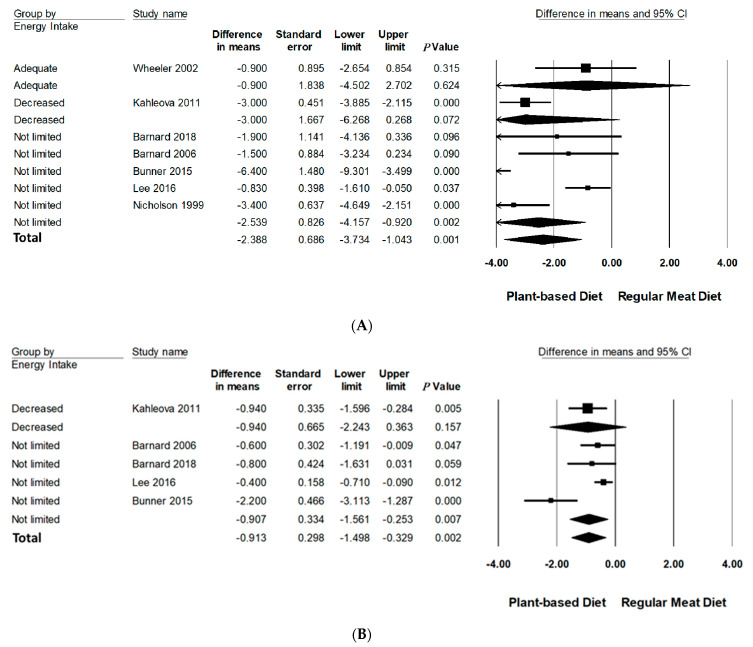
Forrest plots displaying differences in means (MD) and 95% CI for the impact of energy intake of PBDs compared to RMDs on (**A**) body weight (kg) [29,30,31,32,33,34,35] and (**B**) BMI (kg/m^2^) [29,30,32,33,34].

**Figure 4:**
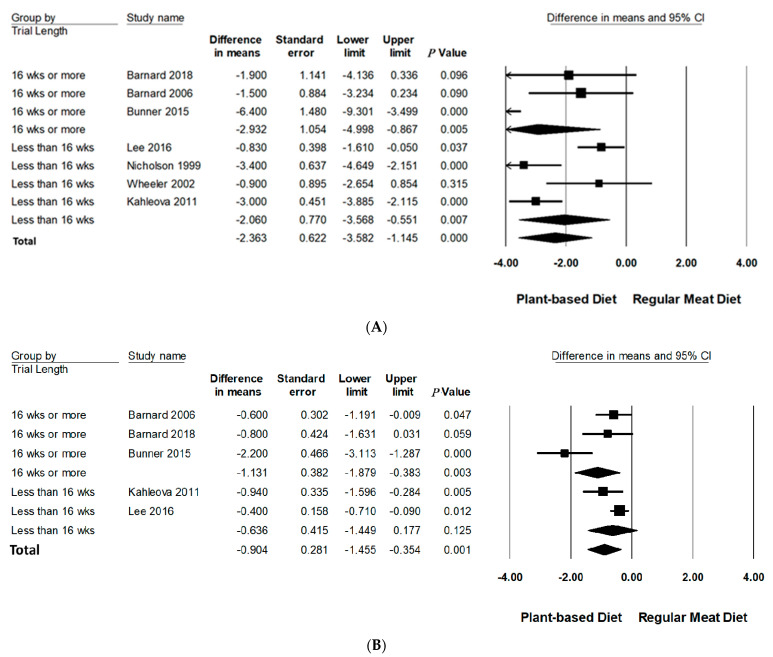
Forrest plots displaying difference in mean (MD) and 95% CI for the impact of trial length (weeks) of PBDs compared to RMDs on (**A**) body weight (kg) [29,30,31,32,33,34,35] and (**B**) BMI (kg/m^2^) [29,30,32,33,34].

Subgroup analyses were performed on body weight and BMI since three or more studies had data available for quantitative analysis (Table 3, Figure 3 and Figure 4 and Figure S3 in Supporting Information Online). All subgroup analysis for WC could not be performed since too few studies had data available. The number of studies in each subgroup are indicated accordingly. Interpretation of these results should be considered exploratory as less than 10 trials were available for each subgroup [24].

#### 3.6.1. Type of PBD

All interventions were categorised as vegan except for one which was categorised as lacto-ovo vegetarian. Compared to RMDs, vegan diets significantly reduced the mean difference of body weight (−2.54 kg, 95% CI: −4.16 to −0.92*, p* < 0.001) and BMI (−0.91 kg/m^2^, 95% CI: −1.56 to −0.25, *p* < 0.01). The one study categorised as lacto-ovo vegetarian did significantly reduce body weight (−3.00 kg, 95% CI: −5.97 to −0.32, *p* < 0.05); however, it did so for BMI.

#### 3.6.2. Energy Intake

Compared to RMDs, interventions that did not limit energy intake statistically significantly reduced mean differences of body weight (−2.54 kg, 95% CI: −4.16 to −0.92, *p* < 0.001) and BMI (−0.91 kg/m^2^, 95% CI: −1.56 to −0.25, *p* < 0.01). Interventions advising adequate and decreased energy intake did not significantly reduce body weight and BMI.

#### 3.6.3. Trial Duration

Trial duration was analysed categorically across two groups: and ≥16 weeks and <16 weeks. Compared to RMDs, studies with a duration of ≥16 weeks reported a statistically significant reduction in mean differences of body weight (−2.93 kg, 95% CI: −5.00 to −0.87, *p* = 0.005) and BMI (−1.13 kg/m^2^, 95% CI: −1.89 to −0.38, *p* < 0.005). Trials conducted for <16 weeks did not significantly reduce BMI but did for body weight, however, to a lesser extent (−2.06 kg, 95% CI: −3.57 to −0.55, *p* < 0.01).

## 4. Discussion

Results from this review suggest vegan dietary patterns significantly reduced body weight, BMI, and WC compared to RMDs in individuals with T2D. Exploratory subgroup analyses demonstrated interventions that advised no limit on energy intake and had a duration of ≥16 weeks were most effective in lowering body weight and BMI.

Various categories of PBDs have been explored in scientific literature for their beneficial effects on lowering body weight in people without diabetes. Recent meta-analyses of RCTs reported consumption of PBDs with no energy restrictions led to significant weight loss in predominately overweight/obese individuals. A meta-analysis of 12 RCTs conducted over a median duration of 18 weeks (ranging from 2 to 24 months) illustrated significant reductions in body weight in those assigned a vegan diet (−2.02 kg) as well as lacto-ovo vegetarian (−1.48 kg) when compared to non-vegetarians [22]. This study did not provide defined methods of categorising PBDs, and therefore, results may not be directly comparable to the current review. The above study is supported by another meta-analysis of 15 RCTs which reported consumption of vegetarian diets for ≥4 weeks lowered body weight (−3.4 kg) in non-diabetic individuals [14]. Vegetarian diets were defined as excluding meat, poultry, and fish and vegan diets defined as excluding animal-derived food products. Lacto-ovo vegetarians were included in analyses; however, no description for inclusion was given. Despite this study providing more delineated definitions than the previous, quantities of dietary consumption were not outlined (e.g., eggs, meat, fish intake) making it difficult to truly ascertain dietary pattern status. A recent publication by our group exploring the effects of PBD on body weight status in Australian women categorised a variety of PBDs as well as frequency of animal product consumption and reported significantly lower body weight in pesco-vegetarians (−10.2 kg) and lacto-ovo vegetarians (−7.4 kg) compared to RMDs [36]. Moreover, increasing weekly frequency of meat intake was associated with increasing body weight, BMI and WC in women who were regular meat eaters. Results from the current review in individuals with T2D are comparable to the findings in individuals without diabetes, however, to a lesser degree, perhaps as a consequence of the small number of studies. Nevertheless, it can be concluded vegan dietary patterns are effective in reducing body weight in overweight and obese adults as well as those with T2D. Larger clinical studies in individuals with T2D and other chronic disease groups which provide detailed and quantifiable definitions for inclusion of all PBD categories are warranted to ascertain the potential varying effects of PBDs on body weight.

BMI was reduced after PBD intervention which is consistent with previously published observational and interventional studies. The EPIC-Oxford cross-sectional study including 37,875 healthy participants compared BMI across four diet groups: regular meat-eaters (no definitions for inclusion); fish eaters (eat fish, no meat); vegetarians (do not eat meat or fish); and vegans (do not eat meat, fish, eggs, or dairy products) using McCance and Widdowson’s food composition tables [37]. Individuals following a vegan diet had the lowest BMI (22.49 kg/m^2^ for men and 21.98 kg/m^2^ for women) compared to ‘regular meat eaters’ (24.41 kg/m^2^ for men and 23.52 kg/m^2^ for women). The authors used categorising techniques that examined a greater range of PBDs which was similar to the current review; however, they did not include lacto-ovo vegetarians nor defined inclusion criterion for ‘regular meat eaters’. Another cross-sectional study involving 55,459 healthy women from the Swedish Mammography Cohort examined risk of overweight and obesity in self-defined PBD groups. These were defined as omnivorous (consume all foods), semi-vegetarian (mostly lacto-vegetarian, sometime consume fish or eggs), lacto-vegetarian (consume no meat, poultry, fish, or eggs), or vegan (consume no meat, poultry, fish, eggs, or dairy products) [38]. Women following a PBD had lower prevalence and risk of being overweight and obesity; in particular, those following a vegan diet had the lowest risk (OR = 0.35) compared to omnivores. Presence of self-reported bias is considerable and PBD definitions did not quantify food intake values.

WC is an effective measure to assess central (visceral) adiposity and is strongly associated with all-cause mortality and cardiovascular mortality with or without adjustment for BMI [39]. This review reported that vegan dietary patterns significantly decreased WC in individuals with T2D. It is noteworthy that only two studies were included in the analyses and should be interpreted as exploratory due to limited scope. In a recent meta-analysis of 40 observational studies, the effects of vegan diets were compared to omnivorous diets on cardiometabolic risk factors and reported significantly lower WC of vegans [40]. No inclusion criteria for PBDs were provided, and the authors noted the definitions of vegan diets varied between studies.

Possible mechanisms behind the effect of PBDs on weight loss include low energy density, glycaemic index (GI), and increased soluble fibre [40,41]. PBDs are generally abundant in wholegrains, fruits, and vegetables rich in phytochemicals, fibre, and antioxidants [8]. It has been suggested that viscous fibre delays gastric emptying and intestinal absorption, therefore enhancing satiety [42]. Fibrous wholegrain foods are often low GI and absorbed slowly resulting in lower postprandial glucose responses and reduced insulin demand [43]. Additionally vegan diets omit major food groups such as meat and dairy which is greatly restrictive compared to RMDs as they do not exclude any food groups. Adhering to a vegan/vegetarian diet was within the exclusion criteria for all studies except one [35]; therefore, dietary patterns do not appear to be matched for restrictiveness.

Exploratory findings from the current review suggest PBD interventions that did not limit energy intake may be more efficacious in reducing body weight and BMI compared to those that controlled dietary intake to meet adequate energy requirements and/or reduced energy intake. In contrast, a meta-analysis previously reported ‘vegetarian diets’ interventions (vegan and lacto-ovo vegetarian) that reduce energy intake were more effective in reducing body weight than trials that did not limit energy intake [22]. Lee et al. 2016 was the only study to report a significant difference in average energy intake between vegan diet intervention and RMD intervention (63 kcal/day difference, *p* = 0.042) which supported no limitations on energy intake [32]. Of the remaining studies which reported post intervention overall energy intake, results between PBDs and RMDs were similar [30,31,32,34]. Moreover, similar energy intake was reported across two previously described meta-analyses which compared vegan and vegetarian diets to conventional diets that limited energy intake over the duration of 6 months [14] and 1.5 years [44]. Results from the current review are cohesive with previous literature and imply PBDs are effective in reducing body weight and BMI irrespective of energy restrictions; however, future studies exploring manipulation of energy intake are required across various types of PBDs to ascertain if this applies to all or only specific types of PBDs.

Only one study which employed a lacto-ovo vegetarian intervention had data available and demonstrated a non-significant reduction in BMI, however, did significantly reduce body weight [29]. The majority of vegan diet interventions individually significantly reduced body weight and BMI, indicating a heavy contribution of vegan diets to the overall pooled analysis in this review. Similar, Huang et al. demonstrated vegan diets as having a greatest weight loss effect than lacto-ovo vegetarian diets in a meta-analysis of 12 RCTs. Furthermore, another meta-analysis reported vegan diets had a more pronounced effect on body weight when compared to lacto-ovo vegetarian diets, yet there were no significant differences between PBD groups in either study [14]. Comparably, results from a five-arm study which used a similar categorising method of PBDs (vegan, vegetarian, pesco-vegetarian, semi-vegetarian) reported vegan diets as having the most pronounced effect on weight loss when compared to omnivores in obese individuals [45]. This trend of vegan diets proving exceedingly effective in reducing body weight than other PBDs is also coherent in studies investigating BMI. The EPIC-Oxford cross-sectional study reported vegans to have a lower BMI than meat-eaters, with a mean difference between groups of 1.92 kg/m^2^ in men and 1.54 kg/m^2^ in women. Overall, results from this review and previous literature are cohesive in reporting vegan diets as most effective in decreasing weight status.

Exploratory analysis of trial length found that trials conducted for ≥16 weeks led to a more pronounced reduction in body weight and BMI compared to trials < 16 weeks. Previous meta-analyses of RCTs have reported increased efficacy in weight reduction in trials with longer durations (6–74 weeks), however, is attenuated after 1 year follow up [14,22]. Interpretation of these results should be considered exploratory as less than 10 trials were available for this subgroup, and therefore, future studies are warranted to delineate both the optimal duration and adherence ability of PBDs for achieving clinically relevant weight loss.

To the best of our knowledge, this study is the first systematic review and meta-analysis comparing the effects of various PBDs with RMD on body weight, BMI, and WC specifically within the T2D population. A similar meta-analysis investigating the effects of vegetarian dietary patterns on CVD in individuals with T2D had comparable results demonstrating a significant reduction in body weight (MD = −2.15 kg), BMI (MD = −0.74 kg/m^2^), and WC (MD = −2.86 cm). Unlike the current study, this study did not differentiate between categories of PBDs [44]. Several limitations of the current review ought to be discussed. Firstly, only a limited number of studies were eligible for inclusion (*n* = 7), and therefore, only exploratory subgroup analyses could be performed. Secondly, there was considerable heterogeneity across studies which could be attributed to methodological variation such as trial duration, lack of standardised categorisation of PBD interventions, and absence of blinding across all studies, which can be attributed to the difficult nature of blinding dietary interventions. Additionally, it should be noted that four of the seven articles shared some of the same authors which may contribute to reporting bias. Considering these limitations, we applied a strict inclusion criterion, only including studies ≥4 weeks and re-categorising various PBDs to pre-defined groups. Moreover, each methodological step was undertaken in duplicate by two independent authors to ensure rigor.

Previous literature has inconsistent methods of defining PBD categories, often neglecting to quantify intake values of animal consumption, creating bias when interpretating comparisons across PBD and RMD. Numerous studies have grouped various PBDs together under one label, e.g., ‘vegetarian’, which creates inconsistency in defining PBDs, misleading translation of findings to clinical practice, and overall contributing to a conflicting pool of literature where the effects of specific types of PBD are diluted. The categorising method defined in this review provides a comprehensive and controlled approach to reporting dietary intake and defining PBDs and has been adapted from a large Australian cohort [46] as well as implemented by our group in a recent paper exploring the effects of PBD on body weight status in Australian women [36]. Physical activity levels and alcohol intake were also inconsistent across studies and were often not accounted for as a confounding factor and/or additional intervention and, therefore, could not be evaluated in the current review. It is pivotal that future studies address these inconsistencies in PBD definitions as well as account for lifestyle factors such as physical activity levels to ensure accurate interpretation of results and translatable dietary recommendations.

In summary PBDs are effective in reducing body weight, BMI, and WC. Vegan diets effectively reduced body weight and BMI. Specifically, a longer study duration ≥ 16 weeks and no limit on energy intake were effective in reducing body weight and BMI. Due to the small size of this review, further clinical trials specifically in T2D are warranted to establish the effects of vegan dietary patterns and other PBDs on body weight and central adiposity. Confirmation of longer intervention durations and unlimited caloric intake alongside PBDs are also warranted to help inform the clinical guidance of these interventions for individuals with T2D. Findings from this study may provide evidence to support the implementation of vegan dietary patterns for assisting in the better management of body weight status in individuals with T2D. Moreover, results may further support research into development of dietary guidelines specific to healthful PBDs for individuals who wish to follow this dietary pattern.

## Figures and Tables

**Table 1:** PICOS criteria for inclusion of studies.

Parameter	Study Selection Criteria
Population	Adults with T2D (≥18 years of age)
Intervention	PBD interventions (‘semi-vegetarian’, ‘pesco-vegetarian’, ‘lacto-ovo vegetarian’, and ‘vegan’) ^1^
Comparator	Dietary patterns including meat (‘regular meat eaters’) ^2^
Outcomes	Weight status (body weight (kg), BMI, WC)
Study design	Randomised controlled trials and prospective cohort studies published in English for ≥6 weeks.

^1^ PBD interventions groups were defined using the following criteria: ‘*Semi-Vegetarian’* if they reported eating 0 or ≤1 time(s) per week beef, lamb, or pork; 0 or ≤1 time(s) per week chicken, turkey, or duck; 0 or ≤1 time(s) per week processed meat and 0 or ≤1 time(s) per week fish or seafood; with a total of ≤1 time(s) per week for the four categories. ‘*Pesco-Vegetarian’* if they reported eating nil beef, lamb, pork, chicken, turkey, duck, or processed meat and ≥1 time(s) per week fish or seafood. ‘*Lacto-ovo Vegetarian’* if they reported eating nil beef, lamb, pork, chicken, turkey, duck, processed meat, fish, or seafood and ≥1 time(s) per week animal-derived foods such as dairy products and/or eggs. ‘*Vegan*’ if they reported excluding all animal flesh and animal-derived foods such as dairy products and eggs. ^2^ Dietary patterns inclusive of meat consumption were defined as ‘*Regular meat-eating diets (RMDs)*’ if they reported eating 0 or ≥1 time(s) per week beef, lamb, or pork; 0 or ≥1 time(s) per week chicken, turkey, or duck; 0 or ≥1 time(s) per week processed meat and 0 or ≥1 time(s) per week fish or seafood; with a total of >1 time(s) per week for the four categories. Abbreviations: PBD, plant-based dietary patterns; BMI, body mass index; T2D, type 2 diabetes; WC, waist circumference.

**Table 2:** Overview of publications included in the systematic review (*n* = 7).

		Participant Characteristics			Treatment Characteristics							
Reference (Quality Score ^a^)	Location, Study Design	Sample Size (% Male)	Mean Age (y)	Baseline wt ^b^	Dietary Intervention Type ^c^	Intervention Delivery ^d^	CHO:PRO:FAT ^e^	Energy Intake ^f^	Total Energy (kcal)(SD) ^j^	Energy Diff (kcal)(SD) ^k^	Length (wks)	Outcomes
Barnard et al. (2018) [30] (+) Intervention Control	United States, parallel	21 (38) 24 (54)	61.0 61.0	34.9 33.0	VeganPortion-controlled	Dietary advice	71:14:1850:21:30	Not limited Decreased	1491 (129) 1332 (85)	−204 (95) * −305 (100) *	20	Wt, BMI
Barnard et al. (2006) [34] (+) Intervention Control	United States, parallel	49 (45) 50 (34)	56.7 54.6	33.9 35.9	Vegan ADA guidelines	Dietary advice	75:15:10 70:20:10	Not limited Decreased ^g^	1425 (427)1391 (382)	−334 (41) * −455 (215) *	22	Wt, BMI, WC
Bunner et al. (2015) [33] (+) Intervention Control	United States, parallel	17 (35) 17 (53)	57.0 58.0	35.9 36.2	Vegan Usual diet	Dietary advice	NR NR	Not limited ^h^ Not limited	-	-	20	Wt, BMI
Kahleova et al. (2011) [29] (+) Intervention Control	Czech Republic parallel	37 (46) 37 (49)	54.6 57.7	35.1 35.0	Lacto-ovo veg DNSG guidelines	Dietary advice	60:15:25 50:20:30	Decreased Decreased	1736 1795	−99 (438) * −37 (837) *	12	Wt, BMI, WC
Lee et al. (2016) [32] (+) Intervention Control	South Korea, parallel	46 (13) 47 (25)	57.5 58.3	23.9 23.1	VeganKDA guidelines	Dietary advice	NR 60:20:25	Not limited Adequate	1409 (549) 1526 (314)	−71 (281) −67 (301)	12	Wt, BMI, WC
Nicholson et al. (1999) [31] (ø) Intervention Control	Unites States, parallel	7 (50) 4 (57)	51.0 60.0	97.7 97.0	Vegan Low-fat diet	Meal supps	75:15:10 60:15:25	Not limited ^i^ Not limited	1409 (549) 1526 (314)	−274 (114) +96 (89)	12	Wt
Wheeler et al. (2002) [35] (+) Intervention Control	Unites States, cross-over	17 (82) 17 (82)	56 56	33.1 33.1	Vegan Animal protein	Meal supps	53:17:30 53:17:30	Adequate Adequate	-	-	6	Wt

^a^ American Dietetic Association’s Quality Criteria Checklist quality score: +, positive; ø, neutral; -, negative. ^b^ Baseline wt only reported when BMI was not available. ^c^ Definitions of interventions previously defined in Table 1. ^d^ Meal supplements (Meal supps) is the provision of some or all meals and food items during the study with no dietary advice. Dietary advice is the provision of personalised nutrition counselling and/or cooking classes. ^e^ Estimated macronutrient composition expresses as a % of total energy intake. If there was no estimation present end of study values for carbohydrates, proteins and fats were calculated from results from nutrient table. ^f^ Decreased energy intake refers to a deficit (≤500 kcal) below energy requirements. Adequate energy intake refers to maintenance of usual energy intake and/or meeting energy requirements. Not limited refers to no limit of energy intake. ^g^ Participants with BMI >25 kg/m^2^ (all participates except 3) were prescribed an energy deficit of >500–1000 kcal. ^h^ The authors advised a limited fat intake to 20–30 g per day with no reference to limits on energy restriction. ^i^ The authors referred to these PBD interventions as ‘low-fat’ with no reference to limits on energy restriction. ^j^ Total energy refers to the energy intake of groups post-intervention. ^k^ Energy difference refers to the within group energy change, significance reported * *p* < 0.05. Abbreviation: ADA: American Diabetes Association; BMI, Body Mass Index; CHO, carbohydrate; Diff, difference; DNSG, Diabetes and Nutrition Study Group; %E, percentage energy; KDA, Korean Diabetes Association; PRO, protein; NR not, reported; veg, vegetarian; wks, weeks; wt, weight; y, years.

**Table 3:** Pooled summary effects and sub-group analysis of PBDs on body weight, BMI, and WC.

Outcome	Subgroup	No. of Intervention Groups	No. Participant (Intervention/Control)	MD (95% CI) ^a^	*p*	Heterogeneity		
						*I* ^2^	P ^c^	%Change ^b^
**Body weight (kg)**	Within PBD group change	7	192	−4.95(−7.34 to −2.55)	<0.001	97.04	<0.001	5.1% ^d^
	Between group differences	7	192/192	−2.35(−3.51 to −1.19)	<0.001	78.63	<0.001	2.4% ^d^
	PBD Type							
	Lacto-ovo veg	1	37/37	−3.00(−5.97 to −0.32)	<0.05	-	-	2.5%
	Vegan	6	155/155	−2.23(−3.60 to −0.87)	=0.001	78.12	<0.001	2.3%
	Energy Intake							
	Not limited	5	138/138	−2.54(−4.16 to −0.92)	<0.001	87.60	<0.005	2.4%
	Adequate	1	17/17	−0.90 (−4.50 to 2.70)	0.62	-	-	-
	Decreased	1	37/37	−3.00 (−6.27 to −0.27)	0.072	-	-	-
	Trial length							
	<16 weeks	4	107/104	−2.06(−3.57 to −0.55)	<0.01	84.91	<0.001	2.1%
	≥16 weeks	3	85/88	−2.93(−5.00 to −0.87)	=0.005	76.46	<0.05	3.0%
**BMI (kg/m^2^)**	Within PBD group change	5	168	−1.87(−2.78 to −0.95)	<0.001	94.95	=0.001	5.4%
	Between group differences	5	168/171	−0.90(−1.42 to −0.38)	<0.001	72.35	<0.01	2.4%
	PBD Type							
	Lacto-ovo veg	1	37/37	−0.94(−2.24 to 0.36)	0.16	-	-	-
	Vegan	4	114/117	−0.91(−1.56 to −0.25)	<0.01	77.95	<0.005	2.4%
	Energy Intake							
	Not limited	4	131/134	−0.91(−1.56 to −0.25)	<0.01	77.95	<0.005	2.4%
	Decreased	1	37/37	−0.94(−2.24 to 0.36)	0.16	-	-	-
	Trial length							
	<16 weeks	2	83/83	−0.64(−1.45 to 0.18)	0.125	-	-	-
	≥16 weeks	3	85/88	−1.13(−1.89 to −0.38)	<0.005	79.79	<0.05	3.0%
**WC (cm)**	Within PBD group change	2	95	−4.23(−6.38 to −2.07)	<0.001	81.01	<0.05	4.3%
	Between group differences	2	95/96	−2.41(−3.72 to −1.09)	<0.001	-	-	2.2%

^a^ Effect sizes expressed as differences in means (MD) and 95% CIs. ^b^ % Change was calculated by dividing the MD by the median baseline level × 100. For parallel studies, the baseline of both the control and test diets was used. For crossover studies, the baseline of the first arm was used. ^c^ *p*-value corresponds to the degree of heterogeneity between studies using *I*^2^ statistic. ^d^ One study (Wheeler 2002 et al.) did not report baseline values therefore excluded in analysis. Abbreviations: BMI, body mass index; Diff, difference; PBD, plant-based diet; Sd, standard; Veg, vegetarian; WC, waist circumference.

## Data Availability

Data described in the manuscript will be made available upon request from the corresponding author.

## Supplementary Materials

The following are available online at https://www.mdpi.com/article/10.3390/nu13114099/s1, Table S1: Quality criteria checklist for included publications. Table S2: PRISMA 2020 Checklist. Figure S1: Funnel plot illustrating publication bias in the studies reporting the effect of PBD on body weight (A) and BMI (B). Figure S2: Leave-one-out sensitivity analysis for body weight (A) and BMI (B). Figure S3: Subgroup analysis for the impact of PBD type on BMI (kg/m^2^).
