# Supplementary material for: Effects of Plant-Based Diets on Weight Status in Type 2 Diabetes: A Systematic Review and Meta-Analysis of Randomised Controlled Trials
Source: Nutrients. 2021 Nov 16;13(11):4099. doi: 10.3390/nu13114099 (PMC8625212; doi:10.3390/nu13114099)
Supplement: Supplementary file 1 [file nutrients-13-04099-s001.zip › nutrients-1420580-supplementary.pdf]

Austin et al. *Effects of Plant-based diets on weight status in type 2 diabetes: A systematic review and meta-analysis of randomised controlled trials*.

SUPPORTING INFORMATION

Table of Contents

Table S1: Quality criteria checklist for included publications **2-7**

Table S2: PRISMA 2020 Checklist. **8-11**

Figure S1: Funnel plot illustrating publication bias in the studies reporting the effect of PBDs on body weight (kg) (A) and BMI (kg/m^2) (B). **12**

Figure S2: Leave-one-out sensitivity analysis for body weight (kg) (A) and BMI (kg/m^2) (B) **13**

Figure S3: Subgroup analysis for the impact of PBD type on BMI (kg/m^2). **14**

Table S1: Quality criteria checklist for included publications.

	**Relevance**				**Validity**										
**Study reference**	**Q 1**	**Q 2**	**Q 3**	**Q 4**	**Q 1**	**Q 2**	**Q 3**	**Q 4**	**Q 5**	**Q 6**	**Q 7**	**Q 8**	**Q 9**	**Q 10**	**Overall**
Barnard et al (2018)	Yes	Yes	Yes	Yes	Yes	No	Yes	Yes	?	Yes	Yes	Yes	Yes	Yes	+
Barnard et al (2006)	Yes	Yes	Yes	Yes	Yes	Yes	Yes	Yes	?	Yes	Yes	Yes	Yes	Yes	+
Bunner et al (2015)	Yes	Yes	Yes	Yes	Yes	Yes	?	?	No	Yes	Yes	Yes	Yes	Yes	+
Kahleova et al (2011)	Yes	Yes	Yes	Yes	Yes	Yes	Yes	Yes	No	Yes	Yes	Yes	Yes	?	+
Lee et al (2016)	Yes	Yes	Yes	Yes	Yes	Yes	Yes	Yes	No	Yes	Yes	Yes	Yes	Yes	+
Nicholson et al (1999)	Yes	Yes	Yes	Yes	Yes	Yes	Yes	No	Yes	Yes	Yes	Yes	Yes	?	**Ø**
Wheeler et al (2002)	Yes	Yes	Yes	Yes	Yes	Yes	Yes	Yes	?	Yes	Yes	Yes	Yes	Yes	+

?: Unclear. +: Positive. Ɵ: Neutral. -: Negative.

<u>Quality Criteria Checklist Questions</u>

<u>Relevance</u>

1. Would implementing the studied intervention or procedure (if found successful) result in improved outcomes for the patients/clients/population group? (NA for some epidemiological studies)

2. Did the authors study an outcome (dependent variable) or topic that the patients/clients/population group would care about?

3. Is the focus of the intervention or procedure (independent variable) or topic of study a common issue of concern to dietetics practice?

4. Is the intervention or procedure feasible? (NA for some epidemiological studies)

Validity

1. Was the research question clearly stated?

1.1 Was the specific intervention(s) or procedure (independent variable(s)) identified?

1.2 Was the outcome(s) (dependent variable(s)) clearly indicated?

1.3 Were the target population and setting specified?

2. Was the selection of study subjects/patients free from bias?

2.1 Were inclusion/exclusion criteria specified (e.g., risk, point in disease progression, diagnostic or prognosis criteria), and with sufficient detail and without omitting criteria critical to the study?

2.2 Were criteria applied equally to all study groups?

2.3 Were health, demographics, and other characteristics of subjects described?

2.4 Were the subjects/patients a representative sample of the relevant population?

3. Were study groups comparable?

3.1 Was the method of assigning subjects/patients to groups described and unbiased? (Method of randomization identified if RCT)

3.2 Were distribution of disease status, prognostic factors, and other factors (e.g., demographics) similar across study groups at baseline?

3.3 Were concurrent controls used? (Concurrent preferred over historical controls.)

3.4 If cohort study or cross-sectional study, were groups comparable on important confounding factors and/or were pre-existing differences accounted for by using appropriate adjustments in statistical analysis?

3.5 If case control study, were potential confounding factors comparable for cases and controls? (If case series or trial with subjects serving as own control, this criterion is not applicable. Criterion may not be applicable in some cross-sectional studies.)

3.6 If diagnostic test, was there an independent blind comparison with an appropriate reference standard (e.g., “gold standard”)?

4. Was method of handling withdrawals described?

4.1 Were follow up methods described and the same for all groups?

4.2 Was the number, characteristics of withdrawals (i.e., dropouts, lost to follow up, attrition rate) and/or response rate (cross-sectional studies) described for each group? (Follow up goal for a strong study is 80%.)

4.3 Were all enrolled subjects/patients (in the original sample) accounted for?

4.4 Were reasons for withdrawals similar across groups?

4.5 If diagnostic test, was decision to perform reference test not dependent on results of test under study?

5. Was blinding used to prevent introduction of bias?

5.1 In intervention study, were subjects, clinicians/practitioners, and investigators blinded to treatment group, as appropriate?

5.2 Were data collectors blinded for outcomes assessment? (If outcome is measured using an objective test, such as a lab value, this criterion is assumed to be met.)

5.3 In cohort study or cross-sectional study, were measurements of outcomes and risk factors blinded?

5.4 In case control study, was case definition explicit and case ascertainment not influenced by exposure status?

5.5 In diagnostic study, were test results blinded to patient history and other test results?

6. Were intervention/therapeutic regimens/exposure factor or procedure and any comparison(s) described in detail? Were intervening factors described?

6.1 In RCT or other intervention trial, were protocols described for all regimens studied?

6.2 In observational study, were interventions, study settings, and clinicians/provider described?

6.3 Was the intensity and duration of the intervention or exposure factor sufficient to produce a meaningful effect?

6.4 Was the amount of exposure and, if relevant, subject/patient compliance measured?

6.5 Were co-interventions (e.g., ancillary treatments, other therapies) described?

6.6 Were extra or unplanned treatments described?

6.7 Was the information for 6.4, 6.5, and 6.6 assessed the same way for all groups?

6.8 In diagnostic study, were details of test administration and replication sufficient?

7. Were outcomes clearly defined and the measurements valid and reliable?

7.1 Were primary and secondary endpoints described and relevant to the question?

7.2 Were nutrition measures appropriate to question and outcomes of concern?

7.3 Was the period of follow-up long enough for important outcome(s) to occur?

7.4 Were the observations and measurements based on standard, valid, and reliable data collection instruments/tests/procedures?

7.5 Was the measurement of effect at an appropriate level of precision?

7.6 Were other factors accounted for (measured) that could affect outcomes?

7.7 Were the measurements conducted consistently across groups?

8. Was the statistical analysis appropriate for the study design and type of outcome indicators?

8.1 Were statistical analyses adequately described the results reported appropriately?

8.2 Were correct statistical tests used and assumptions of test not violated?

8.3 Were statistics reported with levels of significance and/or confidence intervals?

8.4 Was "intent to treat" analysis of outcomes done (and as appropriate, was there an analysis of outcomes for those maximally exposed or a dose-response analysis)?

8.5 Were adequate adjustments made for effects of confounding factors that might have affected the outcomes (e.g., multivariate analyses)?

8.6 Was clinical significance as well as statistical significance reported?

8.7 If negative findings, was a power calculation reported to address type 2 error?

9. Are conclusions supported by results with biases and limitations taken into consideration?

9.1 Is there a discussion of findings?

9.2 Are biases and study limitations identified and discussed?

10. Is bias due to study's funding or sponsorship unlikely?

10.1 Were sources of funding and investigators' affiliations described?

10.2 Was there no apparent conflict of interest

Table S2: PRISMA 2020 Checklist

Section and Topic	Item #	Checklist item	Reported (Yes/No)
**TITLE**			
Title	1	Identify the report as a systematic review.	Y
**BACKGROUND**			
Objectives	2	Provide an explicit statement of the main objective(s) or question(s) the review addresses.	Y
**METHODS**			
Eligibility criteria	3	Specify the inclusion and exclusion criteria for the review.	Y
Information sources	4	Specify the information sources (e.g. databases, registers) used to identify studies and the date when each was last searched.	Y
Risk of bias	5	Specify the methods used to assess risk of bias in the included studies.	Y
Synthesis of results	6	Specify the methods used to present and synthesise results.	Y
**RESULTS**			
Included studies	7	Give the total number of included studies and participants and summarise relevant characteristics of studies.	Y
Synthesis of results	8	Present results for main outcomes, preferably indicating the number of included studies and participants for each. If meta-analysis was done, report the summary estimate and confidence/credible interval. If comparing groups, indicate the direction of the effect (i.e. which group is favoured).	Y
**DISCUSSION**			
Limitations of evidence	9	Provide a brief summary of the limitations of the evidence included in the review (e.g. study risk of bias, inconsistency and imprecision).	Y
Interpretation	10	Provide a general interpretation of the results and important implications.	Y
**OTHER**			
Funding	11	Specify the primary source of funding for the review.	N/A
Registration	12	Provide the register name and registration number.	Y (methods)

From: Page MJ, McKenzie JE, Bossuyt PM, Boutron I, Hoffmann TC, Mulrow CD, et al. The PRISMA 2020 statement: an updated guideline for reporting systematic reviews. BMJ 2021;372:n71. doi: 10.1136/bmj.n71

For more information, visit: http://www.prisma-statement.org/

Section and Topic	Item #	Checklist item	Location where item is reported
**TITLE**			
Title	1	Identify the report as a systematic review.	1
**ABSTRACT**			
Abstract	2	See the PRISMA 2020 for Abstracts checklist.	1
**INTRODUCTION**			
Rationale	3	Describe the rationale for the review in the context of existing knowledge.	1-2
Objectives	4	Provide an explicit statement of the objective(s) or question(s) the review addresses.	1-2
**METHODS**			
Eligibility criteria	5	Specify the inclusion and exclusion criteria for the review and how studies were grouped for the syntheses.	2-3
Information sources	6	Specify all databases, registers, websites, organisations, reference lists and other sources searched or consulted to identify studies. Specify the date when each source was last searched or consulted.	2-3
Search strategy	7	Present the full search strategies for all databases, registers and websites, including any filters and limits used.	2-3
Selection process	8	Specify the methods used to decide whether a study met the inclusion criteria of the review, including how many reviewers screened each record and each report retrieved, whether they worked independently, and if applicable, details of automation tools used in the process.	2-3
Data collection process	9	Specify the methods used to collect data from reports, including how many reviewers collected data from each report, whether they worked independently, any processes for obtaining or confirming data from study investigators, and if applicable, details of automation tools used in the process.	2-4
Data items	10a	List and define all outcomes for which data were sought. Specify whether all results that were compatible with each outcome domain in each study were sought (e.g. for all measures, time points, analyses), and if not, the methods used to decide which results to collect.	3-5
	10b	List and define all other variables for which data were sought (e.g. participant and intervention characteristics, funding sources). Describe any assumptions made about any missing or unclear information.	3-5
Study risk of bias assessment	11	Specify the methods used to assess risk of bias in the included studies, including details of the tool(s) used, how many reviewers assessed each study and whether they worked independently, and if applicable, details of automation tools used in the process.	3-5
Effect measures	12	Specify for each outcome the effect measure(s) (e.g. risk ratio, mean difference) used in the synthesis or presentation of results.	3-5
Synthesis methods	13a	Describe the processes used to decide which studies were eligible for each synthesis (e.g. tabulating the study intervention characteristics and comparing against the planned groups for each synthesis (item #5)).	3-5
	13b	Describe any methods required to prepare the data for presentation or synthesis, such as handling of missing summary statistics, or data conversions.	3-5
	13c	Describe any methods used to tabulate or visually display results of individual studies and syntheses.	3-5
	13d	Describe any methods used to synthesize results and provide a rationale for the choice(s). If meta-analysis was performed, describe the model(s), method(s) to identify the presence and extent of statistical heterogeneity, and software package(s) used.	4-5

Section and Topic	Item #	Checklist item	Location where item is reported
	13e	Describe any methods used to explore possible causes of heterogeneity among study results (e.g. subgroup analysis, meta-regression).	4-5
	13f	Describe any sensitivity analyses conducted to assess robustness of the synthesized results.	4-5
Reporting bias assessment	14	Describe any methods used to assess risk of bias due to missing results in a synthesis (arising from reporting biases).	N/A
Certainty assessment	15	Describe any methods used to assess certainty (or confidence) in the body of evidence for an outcome.	4-5
**RESULTS**			
Study selection	16a	Describe the results of the search and selection process, from the number of records identified in the search to the number of studies included in the review, ideally using a flow diagram.	5
	16b	Cite studies that might appear to meet the inclusion criteria, but which were excluded, and explain why they were excluded.	5
Study characteristics	17	Cite each included study and present its characteristics.	6
Risk of bias in studies	18	Present assessments of risk of bias for each included study.	10-11
Results of individual studies	19	For all outcomes, present, for each study: (a) summary statistics for each group (where appropriate) and (b) an effect estimate and its precision (e.g. confidence/credible interval), ideally using structured tables or plots.	8
Results of syntheses	20a	For each synthesis, briefly summarise the characteristics and risk of bias among contributing studies.	10-11
	20b	Present results of all statistical syntheses conducted. If meta-analysis was done, present for each the summary estimate and its precision (e.g. confidence/credible interval) and measures of statistical heterogeneity. If comparing groups, describe the direction of the effect.	8-14
	20c	Present results of all investigations of possible causes of heterogeneity among study results.	9
	20d	Present results of all sensitivity analyses conducted to assess the robustness of the synthesized results.	9
Reporting biases	21	Present assessments of risk of bias due to missing results (arising from reporting biases) for each synthesis assessed.	N/A
Certainty of evidence	22	Present assessments of certainty (or confidence) in the body of evidence for each outcome assessed.	8-14
**DISCUSSION**			
Discussion	23a	Provide a general interpretation of the results in the context of other evidence.	14
	23b	Discuss any limitations of the evidence included in the review.	16
	23c	Discuss any limitations of the review processes used.	16
	23d	Discuss implications of the results for practice, policy, and future research.	16
**OTHER INFORMATION**			

Section and Topic	Item #	Checklist item	Location where item is reported
Registration and protocol	24a	Provide registration information for the review, including register name and registration number, or state that the review was not registered.	2, 17
	24b	Indicate where the review protocol can be accessed, or state that a protocol was not prepared.	2, 17
	24c	Describe and explain any amendments to information provided at registration or in the protocol.	N/A
Support	25	Describe sources of financial or non-financial support for the review, and the role of the funders or sponsors in the review.	17
Competing interests	26	Declare any competing interests of review authors.	17
Availability of data, code and other materials	27	Report which of the following are publicly available and where they can be found: template data collection forms; data extracted from included studies; data used for all analyses; analytic code; any other materials used in the review.	17

From: Page MJ, McKenzie JE, Bossuyt PM, Boutron I, Hoffmann TC, Mulrow CD, et al. The PRISMA 2020 statement: an updated guideline for reporting systematic reviews. BMJ 2021;372:n71. doi: 10.1136/bmj.n71

For more information, visit: http://www.prisma-statement.org/

A

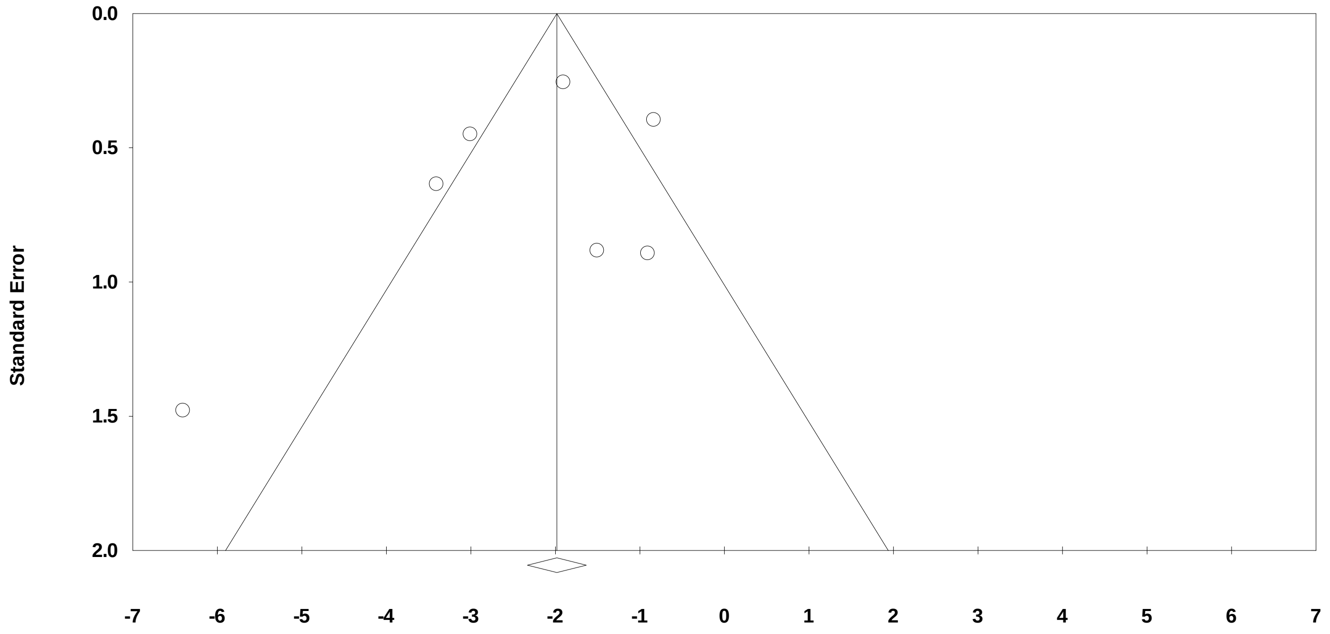

B

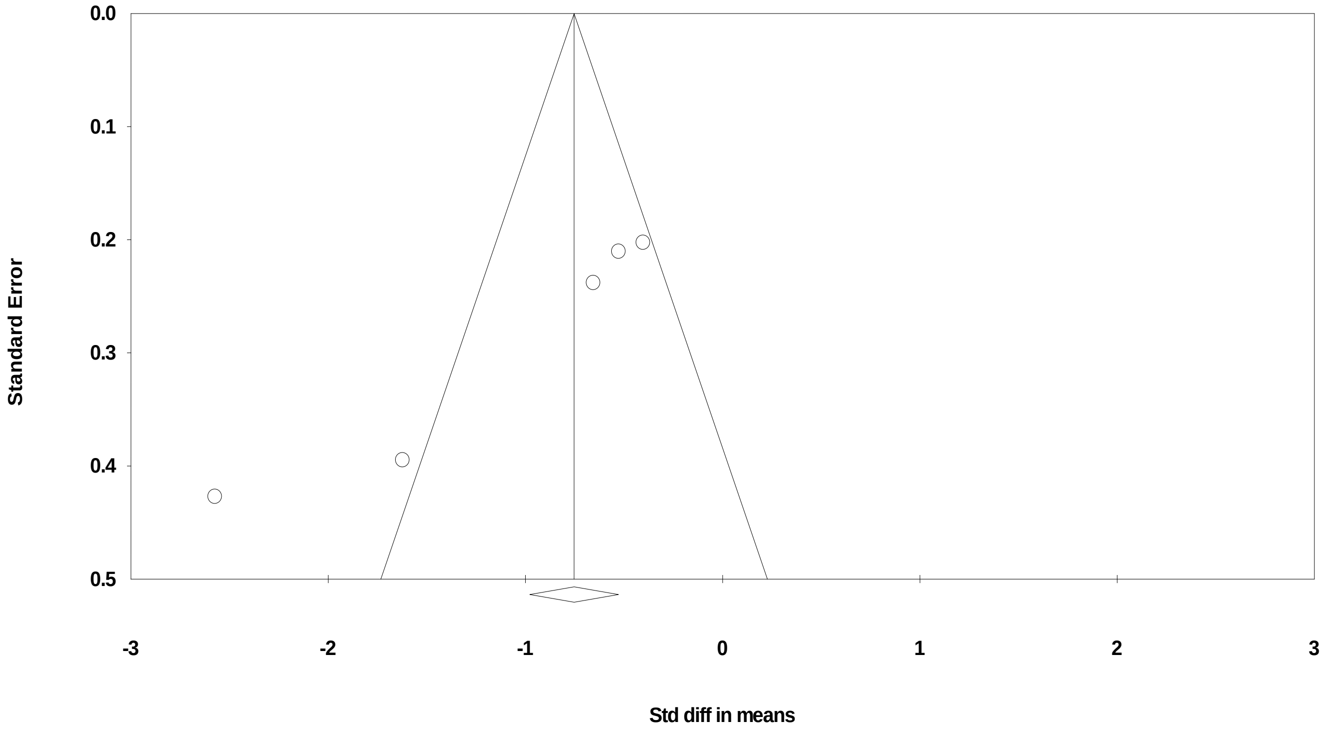

Figure S1: Funnel plot illustrating publication bias in the studies reporting the effect of PBDs on; **(A)** body weight (kg) and **(B)** BMI (kg/m^2).

A

Study name	Point	Standard error	Lower limit	Upper limit	p-Value
Barnard 2018	-2.057	0.245	-2.537	-1.577	0.000
Barnard 2006	-2.003	0.181	-2.358	-1.648	0.000
Bunner 2015	-1.918	0.179	-2.269	-1.568	0.000
Lee 2016	-2.270	0.198	-2.659	-1.881	0.000
Nicholson 1999	-1.863	0.185	-2.226	-1.501	0.000
Wheeler 2002	-2.027	0.181	-2.382	-1.672	0.000
Kahleova 2011	-1.796	0.193	-2.175	-1.418	0.000
**Total**	-1.983	0.178	-2.331	-1.635	0.000

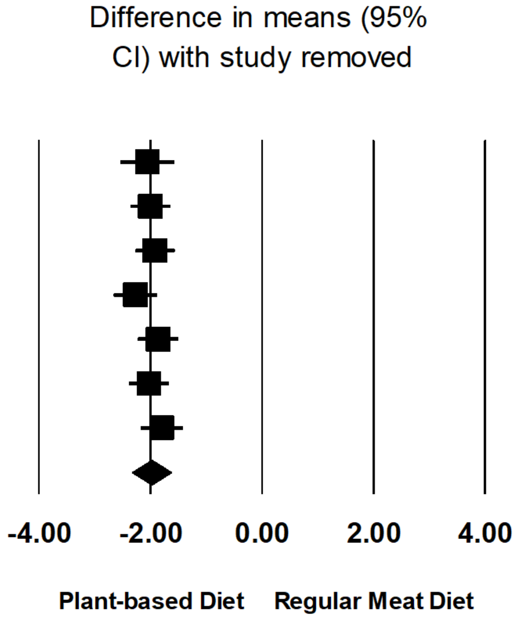

B

Study name	Point	Standard error	Lower limit	Upper limit	p-Value
Kahleova 2011	-0.783	0.131	-1.039	-0.527	0.000
Barnard 2006	-0.918	0.139	-1.190	-0.646	0.000
Barnard 2018	-0.613	0.119	-0.846	-0.380	0.000
Lee 2016	-0.849	0.136	-1.117	-0.582	0.000
Bunner 2015	-0.674	0.120	-0.908	-0.439	0.000
**Total**	-0.753	0.115	-0.978	-0.529	0.000

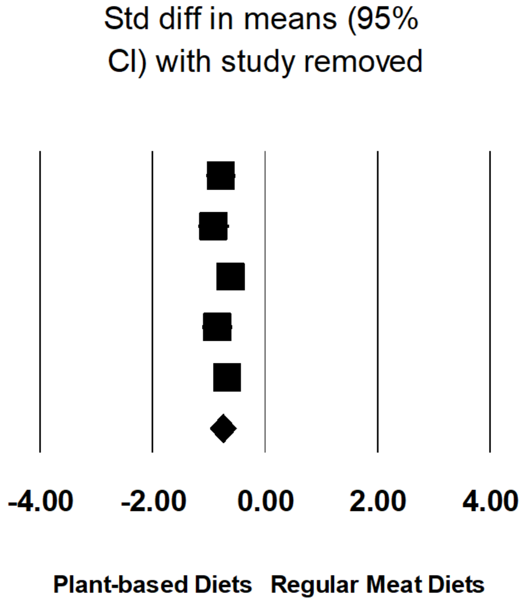

Figure S2: Leave-one-out sensitivity analysis for; **(A)** body weight (kg) and **(B)** BMI (kg/m^2).

A

Group by PBD Type	Study name	Difference in means	Standard error	Lower limit	Upper limit	p-Value
Lacto-ovo	Kahleova 2011	-3.000	0.451	-3.885	-2.115	0.000
Lacto-ovo		-3.000	1.514	-5.968	-0.032	0.048
Vegan	Barnard 2018	-1.900	1.141	-4.136	0.336	0.096
Vegan	Barnard 2006	-1.500	0.884	-3.234	0.234	0.090
Vegan	Bunner 2015	-6.400	1.480	-9.301	-3.499	0.000
Vegan	Lee 2016	-0.830	0.398	-1.610	-0.050	0.037
Vegan	Nicholson 1999	-3.400	0.637	-4.649	-2.151	0.000
Vegan	Wheeler 2002	-0.900	0.895	-2.654	0.854	0.315
Vegan		-2.234	0.695	-3.597	-0.871	0.001
**Total**		-2.367	0.632	-3.606	-1.129	0.000

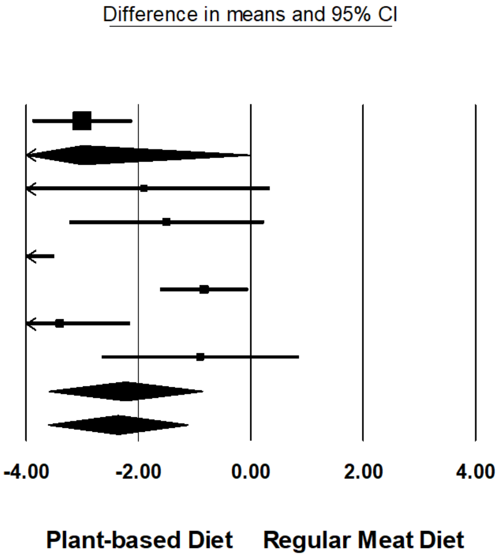

B

Group by PBD Type	Study name	Difference in means	Standard error	Lower limit	Upper limit	p-Value
Lacto-ovo Veg	Kahleova 2011	-0.940	0.335	-1.596	-0.284	0.005
Lacto-ovo Veg		-0.940	0.665	-2.243	0.363	0.157
Vegan	Barnard 2006	-0.600	0.302	-1.191	-0.009	0.047
Vegan	Barnard 2018	-0.800	0.424	-1.631	0.031	0.059
Vegan	Lee 2016	-0.400	0.158	-0.710	-0.090	0.012
Vegan	Bunner 2015	-2.200	0.466	-3.113	-1.287	0.000
Vegan		-0.907	0.334	-1.561	-0.253	0.007
**Total**		-0.913	0.298	-1.498	-0.329	0.002

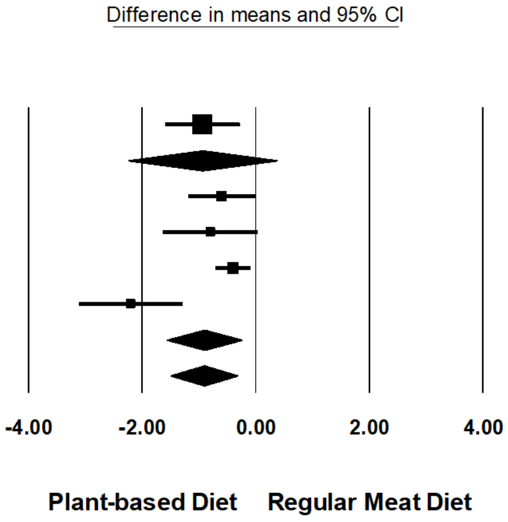

Figure S3: Forrest plots displaying difference in means (MD) and 95% CI for the impact of different PBDs types compared to RMDs on; **(A)** body weight (kg) and **(B)** BMI (kg/m2).
